# Upregulation of Sarcolemmal Hemichannels and Inflammatory Transcripts with Neuromuscular Junction Instability during Lower Limb Unloading in Humans

**DOI:** 10.3390/biology12030431

**Published:** 2023-03-10

**Authors:** Giuseppe Sirago, Julián Candia, Martino V. Franchi, Fabio Sarto, Elena Monti, Luana Toniolo, Carlo Reggiani, Emiliana Giacomello, Sandra Zampieri, Lisa M. Hartnell, Giuseppe De Vito, Marco Sandri, Luigi Ferrucci, Marco V. Narici

**Affiliations:** 1 Department of Biomedical Sciences, University of Padova, 35131 Padova, Italy; 2 Translational Gerontology Branch Longitudinal Studies Section, National Institute on Aging, Baltimore, MD 21224, USA; 3 CIR-MYO Myology Center, University of Padova, 35131 Padova, Italy; 4 Institute for Kinesiology Research, Science and Research Center Koper, 6000 Koper, Slovenia; 5 Department of Medicine, Surgery and Health Sciences, University of Trieste, 34149 Trieste, Italy; 6 Department of Surgery, Oncology and Gastroenterology, University of Padova, 35124 Padova, Italy

**Keywords:** disuse, hemichannels, inflammation

## Abstract

**Simple Summary:**

Skeletal muscles need to be continually active. Physical inactivity, reduced gravity, such as when humans are in space, or aging itself can cause neuromuscular frailty and weakness, causing major diseases. Comprehension of the molecular mechanisms that cause skeletal muscle atrophy can be beneficial to limit such processes and improve human health in different conditions: allowing humans to go into space for a long period, to be inactive for a long period or to age in a better way. When skeletal muscles are chronically inactive, within 5 days and more after 10 days, there is an increase of two molecules, called agrin fragments and neurofilaments, which become highly present in the blood, suggesting neuromuscular instability. The process is accompanied by changes in the membranes of single muscle fibres, the unit that forms our skeletal muscles. They could potentially start to lose important electrolytes and molecules and gradually promote a deleterious process. It has been proved in mice that blocking the appearance of these molecules, called hemichannels, can limit the severity of the loss of muscle mass. Interestingly, these hemichannels also appear during inactivity in humans, opening a possible application for human health and space missions.

**Abstract:**

Human skeletal muscle atrophy and a disproportionate force loss occur within a few days of unloading in space and on Earth, but the underlying mechanisms are not fully understood. Disruption of neuromuscular junction homeostasis has been proposed as one of the possible causes. Here, we investigated the potential mechanisms involved in this neuromuscular disruption induced by a 10-day unilateral lower limb suspension (ULLS) in humans. Specifically, we investigated hemichannels’ upregulation, neuromuscular junction and axonal damage, neurotrophins’ receptor downregulation and inflammatory transcriptional signatures. Biomarkers were evaluated at local and systemic levels. At the sarcolemmal level, changes were found to be associated with an increased expression of connexin 43 and pannexin-1. Upregulation of the inflammatory transcripts revealed by deep transcriptomics was found after 10 days of ULLS. The destabilisation of the neuromuscular junction was not accompanied by changes in the secretion of the brain-derived neurotrophic factor and neurotrophin-4, while their receptor, BDNF/NT growth factors receptor (TrkB), decreased. Furthermore, at 5 days of ULLS, there was already a significant upregulation of the serum neurofilament light chain concentration, an established clinical biomarker of axonal injury. At 10 days of ULLS, other biomarkers of early denervation processes appeared. Hence, short periods of muscle unloading induce sarcolemmal hemichannels upregulation, inflammatory transcripts upregulation, neuromuscular junction instability and axonal damage.

## 1. Introduction

Muscle disuse is the main pathophysiological stress induced by several unloading conditions, such as a sedentary lifestyle, immobilisation, surgery and trauma [1,2]. The exact molecular events underlying disuse-associated atrophy and muscle weakness are not completely understood, and no pharmacological countermeasure is currently available. Hence, a better understanding of the molecular mechanisms that mediate the effects of disuse in the initial phase may reveal novel targets for countermeasures aimed at preventing/reducing the muscle loss induced by prolonged periods of disuse. Recently, studies published by our group and others suggested that sarcoplasmic Calcium (Ca^2+^) leakage and neuromuscular junction (NMJ) impairment are among the early events triggered by a few days of muscle disuse in humans [3,4,5,6,7]. Here, we present an extensive analysis of the molecular processes that may drive the neuromuscular junction impairment that occurs within a few days of disuse in humans. We used the unilateral lower limb suspension (ULLS) model, a condition that mimics physiological changes observed in spaceflight and bed rest (BR) [8]. The model consists of the selective suspension of one leg for a variable duration (10 days in the present study in order to observe the initial processes). ULLS directly affects antigravity muscles and is a well-established model to study the early molecular signatures locally triggered by the absence of mechanical loading, contraction and the excitation–contraction coupling (ECC) cascade. The early stages of the disuse-atrophic program, which follows BR and ULLS in humans, describe the processes of muscle denervation and neural transmission alterations [4,6].

Nerve excision, practised in animal models, induces full-blown denervation and increases sarcolemmal permeability through sarcolemmal hemichannel upregulation [9]. Hemichannels can cause a partial depolarization of the resting membrane potential, promote muscle atrophy and eventually results in inflammasome activation through Ca^2+^ entry or extracellular adenosine triphosphate (ATP) release [9,10,11,12]. Thus, the postsynaptic hemichannels’ appearance may represent a biomarker of altered neuromuscular physiology. Connexin 43 (Cx43) and pannexin-1 (Panx-1) are among the hemichannel isoforms more expressed and investigated in skeletal muscle since they are involved in somatic development [9,13]. Recently, Panx-1 has been proposed as a key player in the hindlimb suspension response of animal models [14], raising the hypothesis that changes in postsynaptic hemichannels may also occur in humans during the early stages of disuse. In particular, we hypothesize that hemichannels could represent new biomarkers of NMJ instability in disuse. Their activation may contribute to the reduced muscle strength and the increased motor unit potential (MUP) complexity observed with ULLS, recently reported by our group in the same cohort [6]. Hence, we hypothesize that NMJ instability and axonal damage, assessed by biomarkers and reported at 10 days of ULLS [6], may occur much earlier. We also posit that the alterations of the NMJ secretome of the brain-derived neurotrophic factor (BDNF) and neurotrophin-4 (NT-4), or of their muscular receptor BDNF/NT growth factors receptor (TrkB), may contribute to NMJ instability with disuse. Indeed, NMJ secretome or the neurotrophins’ receptor alterations may be potentially associated with hemichannels expression. Finally, since hemichannels control gene expression through purinergic receptors inducing muscular inflammation, we also postulate that hemichannels may promote transcriptional reprogramming of the inflammatory response.

## 2. Materials and Methods

### 2.1. ULLS Model and Recruitment

Twelve recreationally active young men (age: 22.1 ± 2.9 years; height: 1.78 ± 0.03 m; body mass: 72.1 ± 7.1 kg) were recruited for this study at the Department of Biomedical Sciences, University of Padova (Italy). The inclusion criteria were 18–35 years of age, a body mass index between 20 and 28 kg m^−2^ and recreational physical activity practice (1 to 3 times per week). The exclusion criteria included sedentary individuals, very active individuals (structured physical activity > 3 times per week), smokers, previous deep venous thrombosis and acute/chronic musculoskeletal, metabolic and cardiovascular disorders. Written informed consent was signed by all the participants.

The participants underwent a 10-day ULLS intervention. A shoe with an elevated sole (50 mm) was provided to fit the non-dominant leg of the volunteers, while the dominant leg was suspended and kept at a slightly flexed position (~15–20 degrees of knee flexion) using straps. The participants were asked to walk with crutches during the ULLS period and to refrain from any movement involving the suspended leg, including driving. A familiarisation session was conducted to provide the participants with sufficient practice in carrying out daily tasks while performing ULLS. For personal reasons, one subject dropped out of the study after the baseline measures.

### 2.2. Human Blood Sampling and Biopsies

Human blood samples and skeletal muscle biopsies from the vastus lateralis (VL) were collected and were in agreement with the submitted and approved ethics. Blood samples were collected at BL, 5 days, and 10 days from the starting date of the ULLS protocol. A small incision was made after anaesthetising the subcutaneous area with lidocaine, and the VL muscle biopsies (100–266 mg) were obtained using a Weil–Blakesley conchotome (Gebrüder Zepf Medizintechnik GmbH & Co. KG, Dürbheim, Germany). The biopsies were then divided into several subfractions depending on the different protocol fates and stored accordingly.

### 2.3. ELISA

Blood samples were collected in Gel Clot Activator tubes (368969, BD Diagnostic, Oxford, UK) and centrifuged at 3000 rpm for 10 min, and then the serum was collected. Quantification of the circulating serum levels of the c-terminal agrin fragment (CAF), brain-derived neurotrophic factor (BDNF) and neurotrophin-4 (NT-4) was performed by kits in duplicates. The adopted kits were the Human Agrin ELISA kit (ab216945, Abcam, Cambridge, UK), Human BDNF ELISA kit (ab212166, Abcam, Cambridge, UK) and Human NT-4 ELISA kit (EHNTF4, Invitrogen, Waltham, MA, USA), respectively. The dilutions for the sample were 1:4 for human agrin, 1:20 for human BDNF and no dilution for human NT-4. The NT-4 level was not detectable in 5 out of 11 subjects at any time point. Quantification was performed by a standard curve with the reconstituted standard provided by the kits and analysed using linear regression or with polynomials, depending on the best fit, as suggested by the guidelines. The absorbance, in accordance with the kit protocol, has been measured by a microplate ELISA reader (Tecan, Infinite F50, Kawasaki, Japan).

### 2.4. Immunostaining

Human muscle biopsies were embedded into OCT (Biognost CF100, Zagreb, Croatia) and fast-frozen in liquid nitrogen-cooled isopentane. Then, serial cryosections of 10 μm were obtained at the cryostat (Leica CM1850, Wetzlar, Germany). For Cx43 and Panx-1, the immunostaining protocol commenced with a pre-chilled acetone fixation at −20 °C. The cryosections were thereafter washed in PBS and incubated in a blocking buffer for 30 min: for Panx-1 and Cx43, 10% normal donkey serum (NDS) and 5% bovine serum albumin (BSA) were used, respectively. The staining was obtained on serial cryosections by incubation with mouse monoclonal primary antibody for Cx43 or Panx-1 in blocking buffer for 2 h at room temperature (RT) and co-incubation with Alexa 568 fluorophore-conjugated goat anti-mouse secondary antibody and wheat germ agglutinin (WGA) for 1 h at RT (Table 1). Serial stained cryosections were then mounted with antifade (ProLong^TM^ Gold Antifade Mountant, P36934), and detection was obtained with a confocal microscope (Leica SP5, Wetzlar, Germany) with a 20× objective at 1024 × 1024 pixels and 10 Hz of resolution. The fluorescence signal was analyzed using the Fiji/ImageJ free software (downloaded at the link https://imagej.net/software/fiji/ accssed on 12 December 2022). In particular, the mean grey value along the sarcolemma of stained myofibers was evaluated manually with the ‘segmented line’ tool and a line width of 5 in a spline fit mode. Generally, the myofibers in the biopsy area appeared with the same intensity. Thus, an average of 20 myofibers was chosen in a blinded way by performing an average among the myofibers of areas at different stain intensities every time it occurred. For NCAM, the immunostaining protocol started with a pre-chilled methanol fixation at −20 °C. The cryosections were thereafter washed in PBS and incubated in 10% fetal bovine serum (FBS) blocking buffer. The staining was obtained by incubation with the primary antibody in 2% goat serum-blocking buffer for 1 h at RT. Then, another blocking buffer was placed (10% goat serum) for 10 min and then incubated with fluorophore-conjugated secondary antibody in PBS for 1 h at RT (Table 1). Serial stained cryosections were then mounted with antifade (ProLong^TM^ Gold Antifade Mountant with 4′,6-diamidino-2-phenylindole dye, D1306, Invitrogen, Waltham, MA, USA), and detection was obtained with a Zeiss microscope connected to a Leica DC 300F camera. NCAM-positive myofibers were counted on the captured images using Image software (https://imagej.net, RRID:SCR_003070 accessed on 18 November 2022) and were expressed as the number of positive myofibers per total number detected in the biopsy area (approximately 400). Antibodies were validated for specificity with a negative control experiment, where no primary antibody was incubated.

### 2.5. Immunoblotting

Human VL muscle fibres were homogenised and solubilized in the Laemmli solution with a pestle (62.5 mM Tris-HCl pH 6.8, 2.3% SDS, 10% glycerol) in the presence of the protease inhibitor at the suggested concentration (Protease Inhibitor Cocktail 100× #5871). The protein concentration was determined by the Folin-Lowry method, using BSA as the standard. The complete separation of the protein profile was obtained using homemade SDS-PAGE (4% acrylamide for stacking and 12% acrylamide for separating). Then, 40 μg of proteins were mixed with the Laemmli solution at 5% β-mercaptoethanol, boiled for 3 min at 90 °C and separated onto the mini-gel system, Mini-PROTEAN^®^ Tetra Handcast System (Bio-Rad, Hercules, CA, USA). Proteins were then transferred to the nitrocellulose membrane on the Mini Trans-Blot^®^ System (Bio-Rad) at 100 V constant for 90 min in a cold transfer buffer containing 25 mM Tris, glycine 192 mM and 20% methanol. The membrane was blocked in orbital shaking with 5% BSA in TBSt pH 7.6 (Tris 0.02 M, NaCl 0.137 M and 0.1% Tween-20). The membrane was incubated ON in a cold room with a primary antibody in a blocking buffer at a specific dilution (Table 1). Subsequently, the incubation took place with HRP-conjugated secondary antibody (Table 1) in 5% BSA TBSt for 2 h at RT. Prior to detection, the membrane was washed again, and the protein signal was visualised by using the enhanced chemiluminescence (ECL Star, EUROCLONE, EMP001005, Milan, Italy) method using a digital imaging system (C-DiGit^®^ Blot Scanner, LI-COR; Image Studio Lite Software analysis system, Lincoln, NE, USA). The molecular weight of the protein bands was detected by comparison with Precision Protein Strep Tactin-HRP Conjugate (161-0380 Bio-Rad) used at the appropriate dilution to detect the Precision Plus Protein WesternC Standards (161-0376 Bio-Rad) on the membrane, co-incubated with secondary antibody. Data were obtained from duplicates of the experiments.

### 2.6. SIMOA Analysis

The SIMOA analysis of light neurofilament was performed on the serum samples (for details about the collection, see the previous ELISA section). The samples were submitted to the SIMOA service offered by Wieslab AB, a Svar Life Sci company, Lundavägen MALMO in Sweden, in accordance with the GLP standards. The SIMOA analysis was performed on a Simoa HD-X Analyzer (PN 10041537) supplied by Quanterix in agreement with the standard protocol suggested by Quanterix. The serum samples were diluted 1:4, and the measurements were obtained in double replicates. The kit used for the reported analysis was the Simoa NF-light Advantage Kit HD-1/HD-X (Item 103186; Lot 502845).

### 2.7. RNA Extraction, Sequencing and Transcriptomic Analysis

The RNA-Seq was performed at the Frederick National Laboratory for Cancer Research Sequencing Facility, NIH. The libraries were made using Illumina’s TruSeq Stranded Total RNA Library Prep protocol. This protocol involves the removal of ribosomal RNA (rRNA) using biotinylated, target-specific oligos combined with Ribo-Zero rRNA removal beads. The RNA was fragmented into pieces, and the cleaved RNA fragments were copied into first-strand cDNA using reverse transcriptase and random primers, followed by second-strand cDNA synthesis using DNA Polymerase I and RNase H. The resulting double-strand cDNA was used as the input to a standard Illumina library prep with end-repair, adapter ligation and PCR amplification performed to provide a library ready for sequencing. The samples were sequenced on the NovaSeq 6000 on an S4 flow cell using paired-end sequencing with a read length of 150 bps. The sequencing quality of the reads was assessed using FastQC (v. 0.11.5), Preseq (v. 2.0.3), Picard tools (v. 2.17.11), and RSeQC (v. 2.6.4) [15,16,17,18]. The samples had 295 to 419 million pass filter reads, with more than 90% of the bases being above the quality score of Q30. In addition, Kraken (v. 1.1) was used as a quality-control step to assess the microbial taxonomic composition [19]. The reads were trimmed using Cutadapt (v. 1.18) to remove the sequencing adapters prior to their mapping to the human reference genome hg38 using STAR (v. 2.7.0 f) in the two-pass mode [20,21]. The expression levels were quantified using RSEM (v. 1.3.0) with GENCODE annotation (v. 21) [22,23]. In order to filter out the low-expressed genes, the filterByExpr function from package edgeR (v. 3.32.1) was used, resulting in 20,555 genes passing through the filter [24]. Subsequently, quantile normalisation was performed using the voom algorithm from the Limma R package (v. 3.46.0), followed by empirical Bayes smoothing of the standard errors to assess the case-vs-control differentially expressed genes adjusted for subject ID (paired analysis) [25,26]. Gene set enrichment analysis was performed using the fgsea package (v. 1.20.0), and the reference gene set collections “Hallmarks” and “Canonical Pathways” (which includes the Reactome, KEGG, WikiPathways, PID and Biocarta gene sets) from the Molecular Signature Database, MSigDB (v. 7.4) [27,28]. A customised implementation in R was developed in-house to add robustness to the GSEA analysis; since GSEA’s enrichment estimates (and statistical significance) are stochastic, our software embeds GSEA in a Monte Carlo algorithm that performs 1000 iterations and chooses significant pathways based on the total statistical ensemble. We have only reported the pathways that appeared significant (based on the adjusted *p*-value < 0.05) in more than 80% of the iterations. We identified nine pathways associated with inflammation and denervation, which appeared significantly enriched in ULLS and were associated with these pathways, and identified a total of 2389 measured genes for downstream analysis (principal component analysis, differential gene expression, volcano plot).

### 2.8. Statistical Analysis and Graphics

A priori power analysis was performed based on the NCAM-positive fibre percentage changes with the G*Power 3.1.9.2 software to determine the appropriate sample size. For an effect size computed based on previous work [4], a required power (1-β) of 0.85 and an error of α = 0.05, the total sample size resulted in 9 subjects. Therefore, the 12 participants recruited in this work represented an appropriate sample, considering potential study drop-outs. The presented data met normality with the Anderson–Darling test, D’Agostino–Pearson test, Shapiro–Wilk test, and Kolmogorov–Smirnov test, unless specified. The data were compared with a two-tailed paired t-test for double comparisons (e.g., between the BSL and LS10), otherwise a one-way ANOVA for multiple time point comparisons (e.g., among BSL, LS5 and LS10) in GraphPad Prism software 9.3.0. was used. The NCAM did not pass normality tests and was evaluated with a non-parametric Wilcoxon test. The bars reported in the graph represent the standard deviation, and the significance reached for the *p*-value was less than 0.05. Graphics were created in GraphPad and Fiji and thus assembled in the GIMP software 2.10.24.

### 2.9. Data Availability

Sequencing data will be deposited in the Gene Expression Omnibus (GEO) repository upon acceptance of this manuscript.

## 3. Results

The ULLS protocol was successfully implemented on 11 participants, and the cross-sectional area (CSA), evaluated by ultrasound at 50% of the VL length, was reduced only on the suspended leg (Figure S1A,B; for Materials and Methods, see Sarto et al., 2022) [6].

### 3.1. Connexin 43 and Pannexin-1 Expression in Human Vastus Lateralis

**Figure 1:**
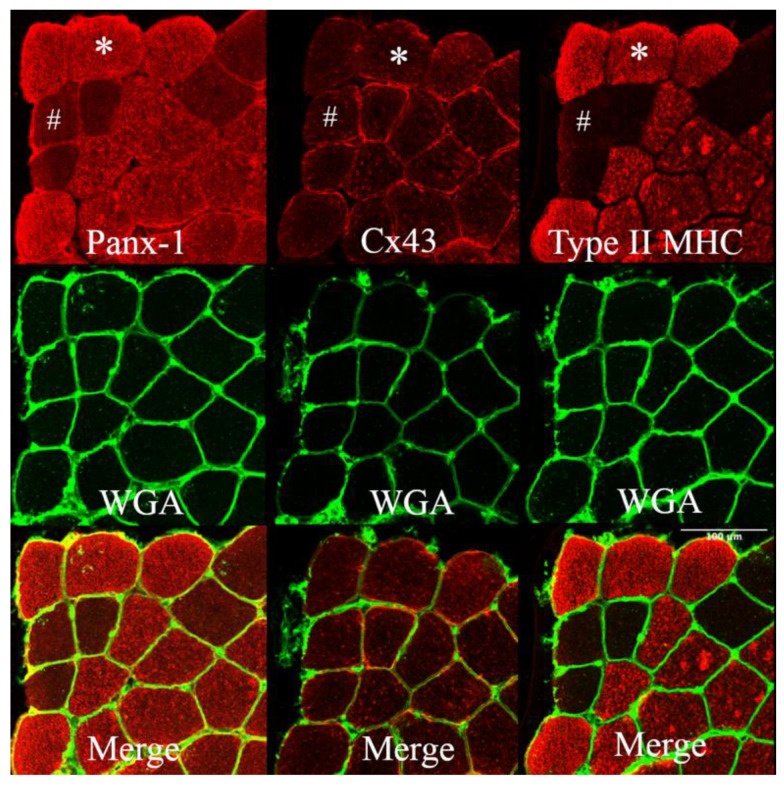
Panels showing Panx-1 and Cx43 immunostaining on human vastus lateralis biopsies at baseline. WGA lectin detects N-acetylglucosamine and N-acetylneuraminic acid (sialic acid) residues, resulting in the staining of the sarcolemma. Staining was obtained on serial cryosections. The staining of Panx-1 appears to significantly overlap with the Type II (IIA + IIX) MHC profile, and the intracellular staining is more prominent in fast muscle fibres. The same fast myofiber is indicated by an asterisk (*) showing Panx-1 intracellular staining. The hashtag (#) indicates the same Type I myofiber with poor Panx-1 intracellular but evident sarcolemma staining. Cx43 shows fainter staining, essentially at the membrane level without fibre-type specificity. Confocal microscope Leica SP5; 20×.

Previous studies in animal models have shown that denervated myofibers express hemichannels at the sarcolemmal level without any expression difference among myofiber types but after denervation with a consistent fast fibres-specific increase in permeability [9,29]. However, in humans, the expression pattern of hemichannels at BSL was different for the VL muscle, in which the expression of Panx-1 was found to be fibre type-specific, while the expression of Cx43 was not. The intracellular Panx-1 staining completely overlapped with the Type II (IIA + IIX) myosin heavy chain (MHC) profile of the serial cryosections (Figure 1), showing that fainter sarcolemma staining also occurs in Type I muscle fibres. This fibre-type expression of Panx-1 is typical of human VL and is shown here for the first time. The same fibre-type specificity of Panx-1 is not reported for any murine skeletal muscles or during denervation [9,29].

### 3.2. Sarcolemmal Upregulation of Connexin 43 and Pannexin-1

**Figure 2:**
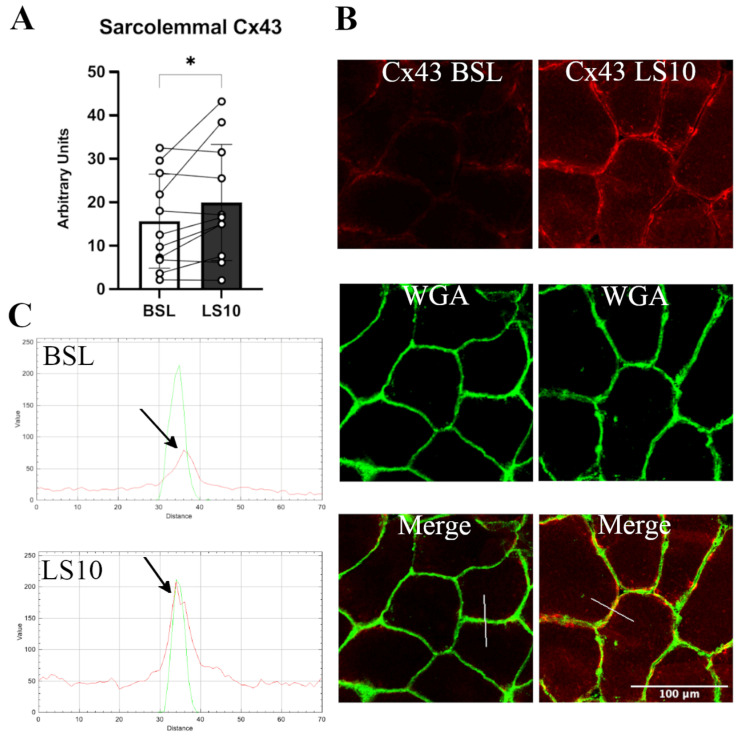
(**A**) Histogram showing average fluorescence units of Cx43 along the sarcolemma. Error bars represent standard deviations. The asterisk (*) indicates statistical significance at *p* < 0.05. (**B**) Typical immunostaining of Cx43 was obtained at baseline and after 10 days of ULLS. In the middle, the WGA signal detects N-acetylglucosamine and N-acetylneuraminic acid (sialic acid) residues of the sarcolemma. In the bottom panels, the merges between the two channels are reported. (**C**) RGB profiles of the immunostaining of Cx43. A line (width 5) has been drawn on the merge images (shown as a white line in the merge images), and RGB profiles were detected along the line. The peaks indicated by the arrows represent the green peak of the WGA signal referring to the sarcolemma fluorescence and the red peak referring to the Cx43 staining. A red peak is present only in the ULLS staining, proving its sarcolemmal upregulation. Intracellular fluorescence intensities in BSL and ULLS panels are different due to increased intracellular staining of Cx43 after ULLS. *n* = 11 male subjects; paired two-tailed *t*-test. Confocal microscope Leica SP5; 20×.

The transcriptomics data allowed us to quantify the normalised mRNA levels of Cx43 and Panx-1, which showed no significant alterations at LS10 (Figure S2). Thus, we further analysed the sarcolemmal protein level of both Cx43 and Panx-1 in immunostaining. For Panx-1, the quantification was fibre-type dependent. The immunostaining for Cx43 showed an increased positivity along the sarcolemma of VL muscle fibres after 10 days of ULLS. The staining was characterised as spotted along the membrane, indicating some preferred regions where Cx43 forms hemichannels, augmented by 28% at LS10 compared with the baseline (Figure 2A, *p* = 0.043). The merging colour confirmed the localization of the membrane signal with the WGA signal and by the plot profile detected along the sarcolemma, with a fluorescence signal peak on the sarcolemma (Figure 2B,C). The comparison of the BSL and LS10 staining obtained on the same slide and acquired with the same exposure, the same pinhole and the same smart gain showed an increase of Cx43 intracellular staining at LS10, as shown in Figure 2B, maybe suggesting an expression on the sarcoplasmic reticulum, as reported for Panx-1 in vitro [30].

**Figure 3:**
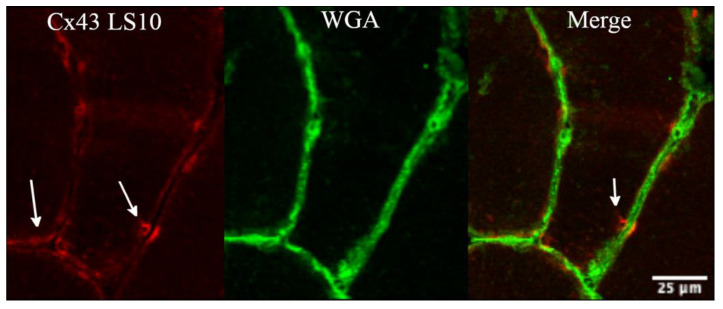
The panel shows detail of the fluorescence signal for Cx43 at higher magnification, obtained along the sarcolemma at 10 days of ULLS. There was no evidence of cluster processes, potentially indicative of gap junction neoformation from fibre to fibre (as indicated by white arrows) with ULLS. Confocal microscope Leica SP5; 20× zoomed in Fiji.

There was no evidence after 10 days of disuse, indicating aggregation between the hemichannels of adjacent fibres to form gap-junctions, as indicated by the higher magnification of the fluorescence signal of Cx43 (Figure 3), which is an isoform able to form gap-junctions.

**Figure 4:**
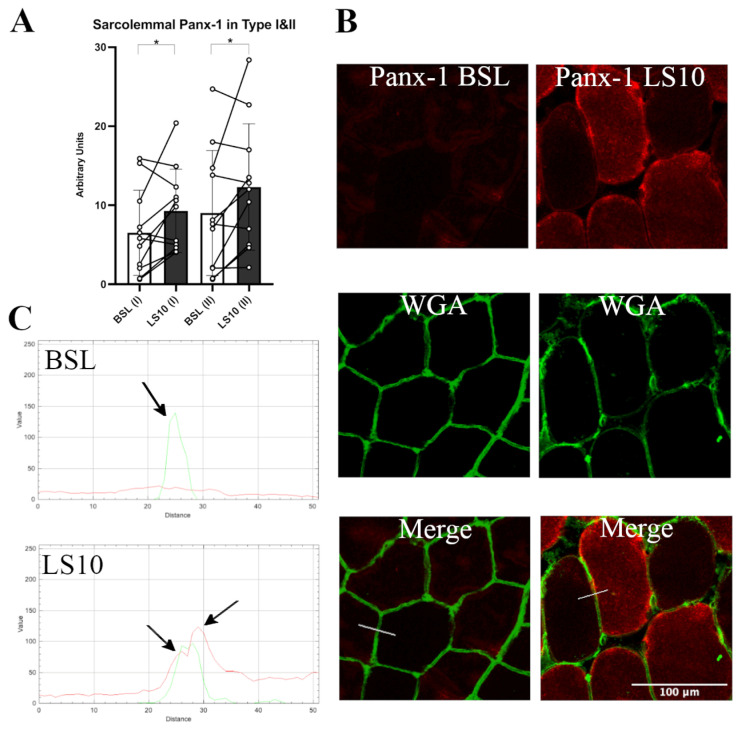
(**A**) Quantification of sarcolemma Panx-1 fluorescence in Type I and Type II (IIA + IIX) myofibers for baseline and after 10 days of ULLS. With (I) and (II) on the x-axis, the corresponding fibre type is indicated. Error bars represent standard deviations. Asterisks (*) indicate statistical significance at *p* < 0.05. (**B**) Typical staining images of Panx-1 for BSL and after 10 days of ULLS. In the middle, the WGA signal detected N-acetylglucosamine and N-acetylneuraminic acid (sialic acid) residues of the sarcolemma. Below are the merges between the two channels. (**C**) RGB profiles of the immunostaining of Panx-1. A line (width 5) has been drawn on the merge images (shown as a white line in the merge images), and RGB profiles were detected along the line. The peaks indicated by the arrows represent the green peak of the WGA signal referring to the sarcolemma fluorescence and the two red peaks (left and right) referring to the Panx-1 staining on the sarcolemma of Type I and Type II myofibers, respectively. A red peak is present only in the ULLS staining, proving its sarcolemmal upregulation. *n* = 11 male subjects; paired two-tailed *t*-test. Confocal microscope Leica SP5; 20×.

The Panx-1 fluorescence signal was evident at both the intracellular space and sarcolemma, confirming the suggestion that this protein may also be expressed in the sarcoplasmic reticulum [30]. We quantified the fluorescence signal for sarcolemma in the fast Type II population (IIA + IIX), and in Type I muscle fibres, comparing the serial cryosections stained for Panx-1 and Type II MHC (Figure 4). We found a 42% mildly significant increase of Panx-1 for Type II (IIA + IIX) fibres and a 36% increase in Type I fibres compared to the baseline (Figure 4A, *p* = 0.048 and *p* = 0.046, respectively). Interestingly, the increased positivity for Panx-1 on the sarcolemma of Type I muscle fibres seemed to be higher than the increase in the intracellular fluorescence, while in Type II, the increase was detected both at the sarcoplasm and intracellular space (Figure 4B). These findings suggest that in Type I fibres, the sarcolemmal upregulation of Panx-1 was preponderant at the sarcolemmal level, while in Type II muscle fibres, an increased expression occurred both at the sarcolemmal and intracellular levels.

### 3.3. Muscle Fibre Denervation Events, Axonal Damage and Agrin Cleavage Processes

**Figure 5:**
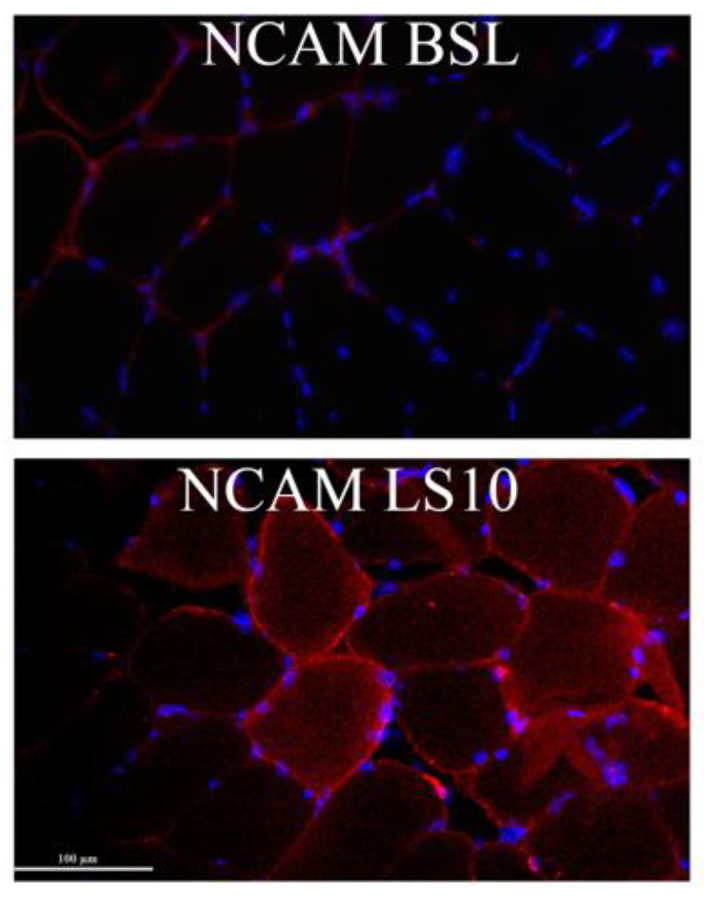
Representative immunohistochemistry images obtained at BSL and 10 days after limb suspension show NCAM-positive myofibers. Zeiss microscope connected to a Leica DC 300F camera, 20×.

In the early stages of denervation occurring during the initial phase of disuse, NCAM is expressed and localised at the sarcolemmal level [4]. As reported by our group in Sarto et al., 2022 [6] and as shown in Figure 5, NCAM-positive muscle fibres augmented after disuse in most subjects.

**Figure 6:**
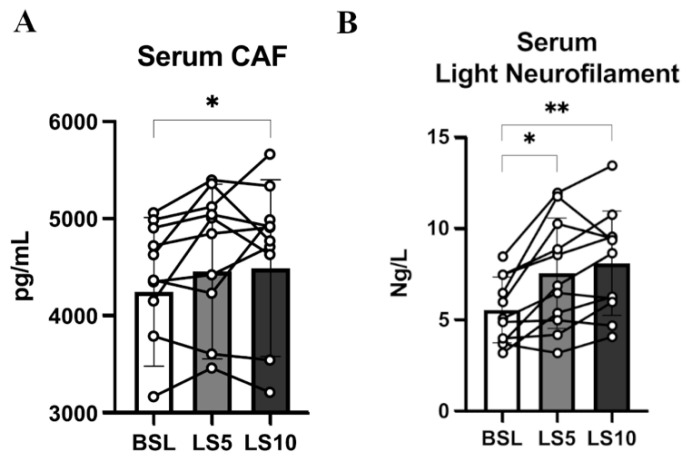
(**A**) Serum level of CAF at BSL, 5 days and 10 days after ULLS. (**B**) Serum level of light neurofilament at BSL, 5 days after ULLS and 10 days after ULLS. Error bars represent standard deviations. Asterisks (*) indicate statistical significance at *p* < 0.05. Two asterisks (**) is for significance at *p* < 0.01. Experiments were performed in duplicates. *n* = 11 male subjects; one-way ANOVA.

To confirm that the initial phase of disuse is accompanied by early denervation processes and, in turn, by damage and instability of the NMJ and axons, we evaluated two circulatory biomarkers of the NMJ and axonal deterioration: CAF and light neurofilaments (NfL), respectively. As already reported by our group, 10 days of ULLS significantly increased the CAF circulatory concentration [6]. To distinguish if the biomarker increases precociously, we measured its concentration 5 days before the end of the protocol. CAF did not increase over the first 5 days (*p* = 0.063), indicated as LS5 (5 days of ULLS) in the graph (Figure 6A), but reached statistical significance only after 10 days of ULLS [6]. No significant changes in the normalised mRNA level of agrin-interacting factors, such as the Muscle-Specific Kinase (MuSK) and the low-density lipoprotein receptor-related protein 4 (Lrp4), were detected in transcriptomics data (Figure S3). Instead, there was an increase of acetyl-colin receptor subunits α1 (AChRα1), AChRβ1, AChRδ and AChRγ, with the latter considered a biomarker of denervation (Figures S4–S6).

The level of NfL increased significantly by 36% after 5 days (*p* = 0.012), suggesting its precocious contribution to NMJ’s instability and behaviour as a reliable biomarker (Figure 6B). NfL increased ulteriorly by 46% after 10 days of ULLS (*p* = 0.001) [6]. Even though the level of NfL could be related to axonal damage at both central and peripheral neuronal levels, these findings suggest that unilateral lower limb suspension caused axonal instability, detectable at the systemic level already at 5 days, possibly directly affecting the neural structures upstream to the NMJ [31,32,33].

### 3.4. Circulating Neurotrophins and Neurotrophin Receptors

**Figure 7:**
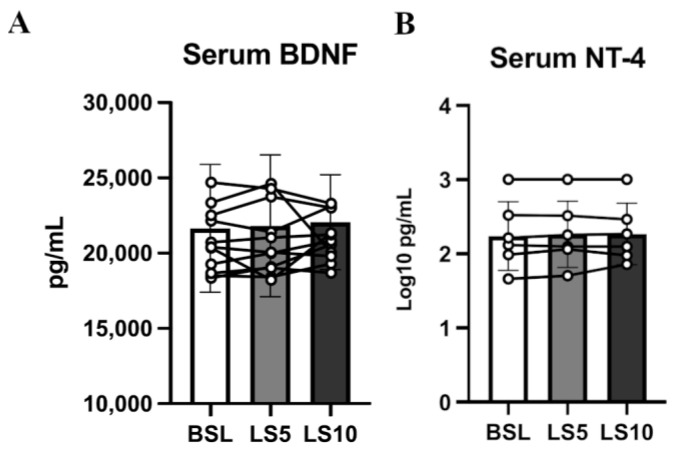
Serum neurotrophin levels were assessed at baseline, after 5 days of ULLS, and after 10 days of ULLS in (**A**) brain-derived neurotrophic factor and (**B**) Neurotrophin-4. Error bars represent standard deviations. All pairwise comparisons between time points were not statistically significant at *p* < 0.05. Experiments were performed in duplicates. *n* = 11 male subjects; one-way ANOVA.

Neurotrophins are important mediators of NMJ homeostasis and favour its stability, especially in muscles mainly composed of fast muscle fibres, such as VL [34]. Animal models suggest that in the presence of E–C coupling events, they act as possible repressors of hemichannels expression [35]. To test whether, during ULLS, there is an involvement of neurotrophins in NMJ homeostasis and hemichannels expression, we analysed the serum levels of the two main neurotrophins, BDNF and NT-4, which share a 50% identity and interact with the same class of receptor, TrkB, which is mainly localised at the NMJ site [36,37]. We found no changes in the serum levels of both neurotrophins (Figure 7).

**Figure 8:**
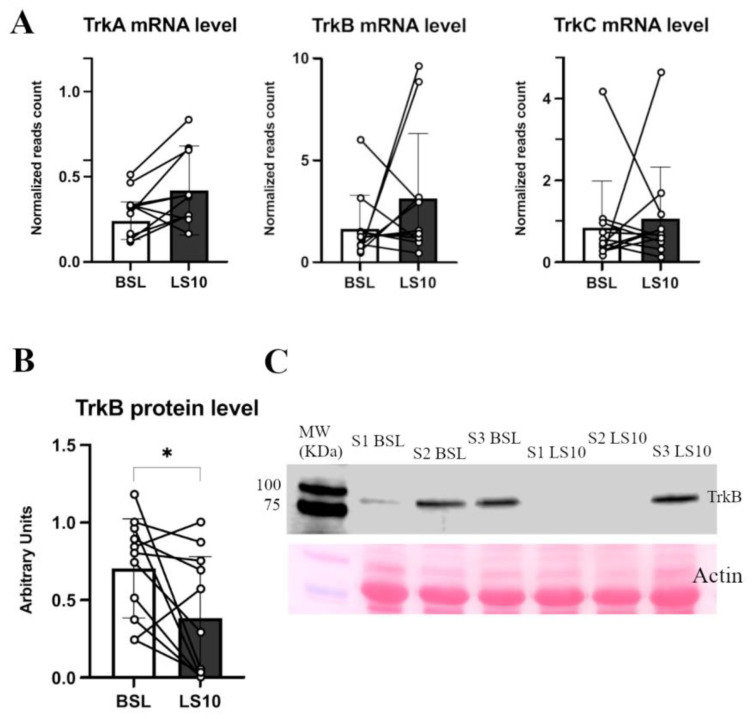
In (**A**), the histograms show TrkA, TrkB and TrkC normalised mRNA levels obtained from transcriptomics data. In (**B**), TrkB protein level was measured in Western blot from duplicate experiments, of which (**C**) shows a representative blot. Error bars represent standard deviations. Asterisks (*) indicate statistical significance at *p* < 0.05. Experiments were performed in duplicates. N = 11 male subjects; paired two-tailed t-test for TrkA mRNA and TrkB protein; paired Wilcoxon test for TrkB and TrKC mRNA.

No significant changes at the transcriptional level in the TrkA, TrkB and TrkC mRNA levels were found, as reported by the transcriptomics data (Figure 8A). However, the protein level of their receptor, TrkB, was halved (−45%; *p* = 0.035) (Figure 8B,C and Figures S7–S9).

### 3.5. Transcriptomics Analysis of ULLS vs. Baseline Differences

**Figure 9:**
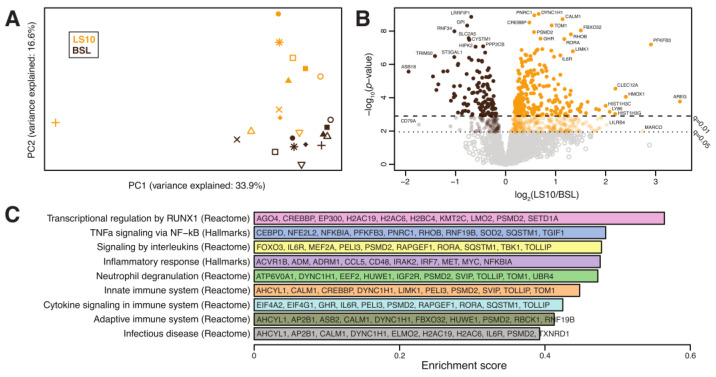
(**A**) Principal component analysis of the transcriptomes from 11 subjects at baseline and after 10 days of ULLS. (**B**) Volcano plot showing differentially expressed genes between ULLS and baseline. Genes with the most significant and/or largest fold-change differences are annotated. (**C**) Differentially enriched pathways. For each pathway, we display in alphabetical order the 10 most significant differentially expressed genes (selected amongst GSEA leading edge genes with differential gene expression q < 0.05). *n* = 11 male subjects; gene set enrichment analysis.

Biomarkers of the initial denervation processes and sarcolemmal hemichannels upregulation were the main hallmarks of the cellular changes induced locally by disuse. To test if sarcolemmal hemichannels upregulation was associated with inflammatory transcriptional reprogramming, we performed a differential gene expression analysis based on total RNA-Seq to focus on the differences between the baseline and 10 days of ULLS on the VL. After pre-processing, normalisation, and filtering steps (see Materials and Methods section for details), the expression of 20,555 genes on 11 subjects was utilised for our downstream analyses. Globally, a significant shift in the overall expression was observed between the baseline and ULLS, as evidenced by principal component analysis. Among the overall pathways, we identified nine pathways associated with inflammation and denervation that appeared significantly enriched in ULLS (described below). Associated with these pathways, we identified a total of 2389 measured genes. Principal component analysis based on these genes showed a significant shift in the overall expression between the baseline and LS10 (Figure 9A). Moreover, at the individual gene level, we found 688 (29%) genes differentially expressed at FDR-adjusted q < 0.05, of which 474 (20%) were over-expressed, and 214 (9%) were under-expressed in LS10 relative to the baseline (Figure 9B). With a more stringent cut-off at q < 0.01, we observed 242 (10%) over-expressed and 120 (5%) under-expressed genes in LS10 relative to the baseline. The nine pathways associated with inflammation and denervation that appeared significantly enriched in LS10 are shown in Figure 9C. The ten most significant differentially expressed genes are also displayed for each of them. Notably, and in agreement with the increased CAF levels and NCAM-positive fibres with ULLS, we observed the enrichment of genes involved in transcriptional regulation by runt-related transcription factor 1 (RUNX1). This gene encodes a DNA-binding protein required to limit the severity of the atrophic program activated by disuse and denervation, as shown in animal models [38]. In our human disuse model of ULLS, the RUNX1 pathway appears to be activated at the transcriptional level, supporting the NMJ instability hypothesis during ULLS. Moreover, the hemichannels contribute to the ‘NACHT, LRR and PYD domains-containing protein 3′ (abbreviated in NLRP3) inflammasome activation through a transcription factor called the nuclear factor kappa-light-chain-enhancer of activated B cells (abbreviated to NF-κB), whose activation drives the expression of proinflammatory interleukins, such as interleukin-6 (IL-6) and tumour necrosis factors (TNFs) [9].

Interestingly, our analysis identified the hallmark pathways of TNF alpha signalling via NF-κB, the inflammatory response, innate and adaptive immune systems, infectious disease and processes, such as neutrophil degranulation and cytokine signalling, among those significantly enriched in LS10 (Figure 9C). Among the ten most significant differentially expressed genes, some are shared between the different sets of pathways used for the analysis. The function of these genes and the literature related to skeletal muscle physiology reveals that LS10-enriched genes are involved in IL6 signalling, such as CCAAT/enhancer-binding protein (CEBP) and interleukin-6 receptor (IL6R); TNF signalling, such as NF-kappa-B inhibitor alpha (NFKBIA) or inflammatory molecules, such as CD48 antigen, interleukin-1 receptor-associated kinase-like 2 (IRAK2) and interferon regulatory factor 7 (IRF7).

## 4. Discussion

As recently reported by our group, in response to unloading, the quadriceps’ maximum voluntary contraction of the suspended leg decreased by 29.3%; this was only partly due to a decrease in skeletal muscle mass (4.5%) [6] and partly to reduced voluntary activation (6%) [6]. As a result, a marked reduction in muscle force per unit area (28%) was observed at the end of the suspension period [6]. Atrophy was induced only in the suspended limb, as confirmed by the CSA, which was reduced only in the suspended limb. We previously proposed that NMJ instability, muscle fibre denervation, sarcoplasmic reticulum Ca^2+^ dynamics and altered MUP properties may account for this reduction in intrinsic muscle force capacity [4,6]. Here, we provide novel empirical evidence on the molecular mechanisms concerning the electrophysiological alterations and NMJ instability induced by unloading. We highlight that the axonal damage and NMJ destabilisation were reported at 10 days [6], as an early event may already occur at 5 days of ULLS. At 10 days of ULLS, this is accompanied by the upregulation of the sarcolemmal Cx43 and Panx-1 hemichannels, marked enrichment in inflammatory pathway transcripts and a reduced level of muscular neurotrophins receptor TrkB in response to ULLS in a population of young men.

### 4.1. Circulatory and Transcriptional Signatures of Muscle Denervation upon Disuse

The NMJ is an essential site for the E–C coupling process and is thus one of the first affected structures in response to disuse. When disuse is prolonged, NMJ instability may contribute to the activation of the atrophic program [3,4,5,6]. Thus, a better understanding of the causes and players involved in NMJ instability may clarify the pathophysiological processes underlying disuse atrophy [39]. In this ULLS experiment, evidence of NMJ instability and axonal damage was suggested by the increased serum concentrations of CAF and NfL in response to 10 days of unloading, as recently reported by our group in the same cohort [6]. Evidence of the initial and partial denervation processes was highlighted by the increase in NCAM-positive myofibers, the AChRγ subunit mRNA and by the increase in the transcriptional regulation induced by RUNX1 activation—the DNA-binding protein required for limiting the atrophic program induced by denervation [38]. Muscular AChRγ, RUNX1 and NCAM are typically associated with denervation, and their expression may suggest suffering of muscle fibres under disuse.

Agrin is a proteoglycan deposited at the NMJ cleft that is cleaved upon the action of the protease neurotrypsin, which, after the cleavage, releases into the blood CAF. The origin and the role of the augmented cleavage of agrin during the early stages of disuse are not well understood. The parallel increase of NCAM—the glycoprotein that drives the reinnervation processes at the sarcolemmal level—supports the idea that attempts of reinnervation rapidly follow the process of denervation; resembling NMJ instability might be evoked by a reduction in the muscle-nerve crosstalk, as corroborated by the reduced muscle protein content of the TrkB neurotrophin receptor. The neural impairment associated with disuse also seems to affect the axonal integrity as a precocious event. In fact, an increased serum concentration of NfL was found after only 5 days of ULLS but without the possibility of discerning between central and peripheral neurons. Higher levels of NfL after 10 days of ULLS have already been reported [6]. The present findings suggest that unloading triggers early instability of the NMJ and of the axons—events that may precede or accompany the activation of the atrophic program when disuse is prolonged. Furthermore, at 5 days of ULLS, the precocious axonal damage could even exacerbate NMJ instability and reduce the ability to generate muscle force after a period of disuse, given the mutual influence between the axon and NMJ reported in disease [40]. This seems consistent with an increased mean inter-discharge interval (IDI mean) obtained from intramuscular electromyography recordings after 10 days of ULLS [6].

### 4.2. Sarcolemmal Hemichannels Upregulation and Inflammatory Transcriptional Signatures Response upon Muscle Disuse

The precise origin of the vulnerability of the NMJ and the axon upon disuse is presently unclear, but some speculations can be made based on the upregulated hemichannels, which normally accompany skeletal muscle development but almost disappear with the maturation of the NMJ [41]. The sarcolemmal upregulation of Panx-1 and Cx43 also supports the initial processes of denervation since the de novo expression of Cx43 and the sarcolemmal upregulation of Panx-1 have been reported in unilateral hindlimb denervated animal models [9,29]. Interestingly, in the same animal models, the knock-out of Panx-1 or Cx43 prevents a decrease in the myofibers’ cross-sectional area; the knock-out also prevents the transcription and activation of the inflammasome complex, even in the presence of unilateral hindlimb denervation [9]. Thus, hemichannels are typically expressed during full-blown denervation of animal models; and accompanying the initial state of denervation occurring with ULLS in humans may be an essential part of the preparation for the atrophic program and maybe of the inflammasome activation induced by unloading. The ability of hemichannels to mediate inflammasome activation could be related to the Ca^2+^ influx and ATP efflux, both insults concurring with the transcription and activation of the inflammasome complex [42]. Interestingly, a Ca^2+^ unbalance and extracellular ATP release upon disuse have been reported both in animal and human models of disuse [4,14]. Notably, our advanced analysis of deep transcriptomics data detected clear signs of the inflammatory transcriptional reprogramming of muscle fibres after 10 days of ULLS, which might be mediated by the purinergic signal under hemichannels control. Muscle fibres showed activation of almost all the transcriptional inflammatory pathways. The susceptibility of muscle fibres to inflammation through the NLRP3 inflammasome activation and the release of cytokines, interleukins and TNF agrees with the endocrine properties of skeletal muscle. Notably, in murine myotubes, NLRP3 activation may occur independently from the immune system’s involvement and result in reduced myotube diameter and growth, typical signs of disuse atrophy [43].

We are aware that the present article cannot define the exact physiological role of the increased Panx-1 and Cx43 expression in ULLS. However, these findings possibly suggest that changes in their transcriptional coupling through purinergic receptors may contribute to the activation of inflammatory transcriptional pathways during disuse, as confirmed in the animal model of denervation [9]. Furthermore, sarcolemmal hemichannels upregulation and their possible opening during E-C coupling events may also contribute to some of the motor units’ electrophysiological alterations, which were observed with ULLS in our parallel study [6]. Indeed, changes in the hemichannels’ dynamics could impair the propagation of electrical impulses along the sarcolemma and result in an increased MUP complexity due to altered membrane permeability. This hypothesis seems confirmed by the recent observations by our group, where MUP complexity increases after ULLS and may partially explain the disproportionate force loss observed with disuse [6].

### 4.3. Neurotrophins’ Secretome, TrkB Receptor and Cxs Expression upon Muscle Disuse

It has been proposed that the expression of Cxs in skeletal muscle could be regulated by neurotrophins and agrin-AChR signalling and that it can be finely linked to NMJ homeostasis [35]. We found unchanged levels of circulatory BDNF and NT-4 but halved muscle protein levels of their muscular receptor TrkB, which suggests a possible involvement of depressed neurotrophin receptors in mediating Cxs upregulation due to disuse. The involvement of BDNF and the TrkB axis in influencing NMJ homeostasis is emerging as fundamental for muscles composed mainly of fast fibres, such as tibialis anterior or VL [34]. The neurotrophin receptor decrement seems not dependent on transcriptional deregulation, where transcriptomics revealed an unchanged mRNA level, maybe suggesting a decoupling between transcription and translation of the neurotrophin receptor proteins due to the disuse condition. Such a decoupling between transcription and translation is not new during unloading conditions since it has also been observed in the changes of contractile proteins of murine models subjected to microgravity conditions [44]. Sarcolemmal Cxs upregulation could be due to an imbalanced agrin signalling since motor-neuron agrin may be depressed after disuse due to an increased release of circulatory CAF. However, the transcriptomics revealed unchanged MuSK and Lrp4 mRNA levels, and both are involved in agrin signalling. In contrast, a hypothetical compensatory effect is evocated by the upregulation of AChRα1, β1 and δ subunits mRNA levels, maybe due to the possible motor-neuron agrin decrement.

**Figure 10:**
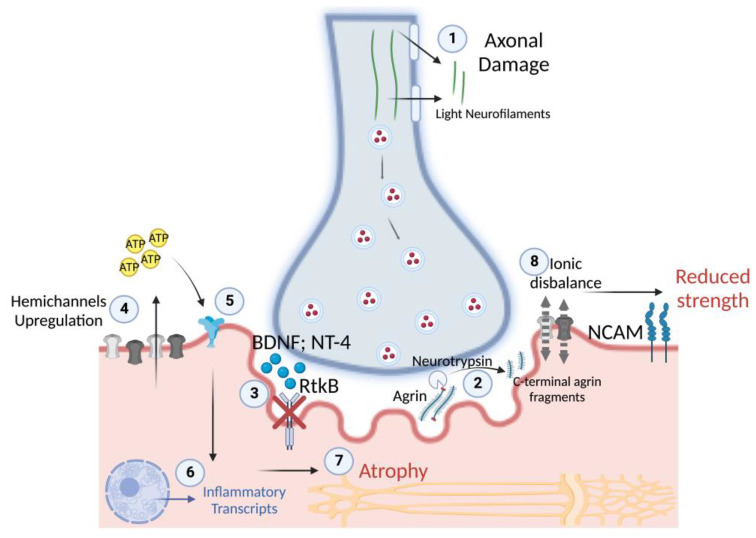
Overview of the potential physiological mechanisms during the early stages of disuse in humans. (1) After 5 days of disuse, the release of light neurofilaments suggests axonal damage, which may progressively cause NMJ instability (2) through the cleavage of agrin and the expression of NCAM, which show significance at 10 days of disuse. NMJ instability is associated with (3) reduced RtkB neurotrophin receptors for BDNF and NT-4, (4) which may be responsible for the marked sarcolemmal hemichannels’ upregulation after 10 days of disuse. In turn, hemichannels may start to release ATP with paracrine properties, (5) acting on purinergic receptors, (6) resulting in the transcription of inflammatory transcripts and (7) in muscle atrophy, with the progression of disuse. Indeed, (8) sarcolemmal hemichannels upregulation may cause ionic disbalance along the sarcolemma, resulting in unstable membrane potential at rest and a reduced capacity to generate efficacious excitation-contraction coupling cascades, resulting in reduced muscle strength.

The unchanged level of BDNF and NT-4 neurotrophins may even be detrimental in the context of the NMJ’s early changes associated with disuse. In fact, BDNF and NT-4 prevent excessive AChRs clustering at the NMJ in an agrin-independent manner [45]. It means that the presence of agrin may limit the effects of BDNF and NT-4 on AChRs clustering to modulate the NMJ maturing process [45]. However, in the presence of NMJ instability and CAF release, the unchanged levels of BDNF and NT-4 could further affect AChRs clustering in adverse conditions. The release of axonal NfL, reported already at 5 days of ULLS, may also be related to the circulatory neurotrophin concentrations. In fact, BDNF and NT-4 expression may even be detrimental during peripheral axon repair and NMJ regeneration, as reported in the literature [37]. Thus, the unchanged level of these neurotrophins in the presence of NMJ instability and axonal damage during ULLS may be deleterious, inhibiting the repair processes of the peripheral axon and NMJ and exacerbating the condition. An explanation of the physiological implications potentially emerging from our study is reported in Figure 10 as follows.

## 5. Conclusions

Here, we show for the first time that an initial state of disuse is characterised by sarcolemmal hemichannels Panx-1 and Cx43x upregulation, inflammatory signatures, and reduced neurotrophins’ TrkB receptor in a context of NMJ instability and axonal damage, which may lead to the activation of the atrophic program if disuse is protracted. The modulation of hemichannels expression, or their sarcolemmal relocation during skeletal muscle disuse, possibly represents a potential molecular target to limit the impact of inflammation and neuromuscular damage caused by muscle unloading, as already proposed in the animal models of nerve excision [9,29].

## 6. Limitations

The authors recognise some limitations of the present study: (i) the inability to provide functional experiments on the opening of Cx43 and Panx-1 channels; (ii) the absence of experiments confirming inflammasome activation. However, the aim of the present study is not to establish systemic or local inflammation but to establish the early molecular events that could result in the activation of the atrophic program with the progression of muscle disuse.

## Figures and Tables

**Table 1:** Antibodies. The table includes primary and secondary antibodies, as well as dilutions, adopted in the study.

Antibody	Code	Type	Dilution	Reaction Type
Anti-Connexin 43	sc-271837	Mouse Monoclonal	1:50	Unconjugated
Anti-Pannexin 1	sc-515941	Mouse Monoclonal	1:30	Unconjugated
Type II MHC	M 4276 Sigma	Mouse Monoclonal	1:1000	Unconjugated
Goat anti-Mouse	A-11031 Invitrogen	Alexa 568	1:300	Alexa 568
WGA	W11261 TF	Alexa 488	1:1000	Alexa 488
NCAM	AB5032 Chemicon	Rabbit Polyclonal	1:200	Unconjugated
Goat anti-Rabbit	A-11012 Invitrogen	Alexa 594	1:200	Alexa 594
Anti-TrkB	AB9872 Millipore	Rabbit Polyclonal	1:5000	Unconjugated
Goat anti-Rabbit	ab205718 Abcam	Goat Polyclonal	1:10,000	HRP

## Data Availability

The data presented in this study are openly available in https://osf.io/cz254/?view_only=938b4b38694c416bbd4b95a8ff0f8155 accessed on 12 December 2022.

## Supplementary Materials

The following supporting information can be downloaded at: https://www.mdpi.com/article/10.3390/biology12030431/s1, Figure S1: histograms showing a cross-sectional area of vastus lateralis at 50% of muscle length, control leg (A) and suspended dominant leg (B); Figure S2: histograms showing normalised read counts of the mRNA levels of connexin43 (A) and pannexin-1 (B) at baseline and after 10-day ULLS, obtained from transcriptomics data; Figure S3: histograms showing normalised read counts of the mRNA levels of MuSK (A) and Lrp4 (B), obtained from transcriptomics data; Figures S4–S6: histograms showing normalised read counts of the mRNA levels of acetyl-choline receptor subunits α1 (S4A), β1 (S4B), δ (S5A), ε (S5B) and γ (S6); Figures S7–S9: original Western blot of TrkB for each subject.
