# Supplementary material for: Upregulation of Sarcolemmal Hemichannels and Inflammatory Transcripts with Neuromuscular Junction Instability during Lower Limb Unloading in Humans
Source: Biology (Basel). 2023 Mar 10;12(3):431. doi: 10.3390/biology12030431 (PMC10044797; doi:10.3390/biology12030431)
Supplement: Supplementary file 1 [file biology-12-00431-s001.zip › biology-2208822-supplementary.pdf]

**Supplementary Material of**

# Upregulation of sarcolemmal hemichannels and inflammatory transcripts with neuromuscular junction instability during lower limb unloading in humans

Giuseppe Sirago[1,*]; Julián Candia[6]; Martino V. Franchi[1]; Fabio Sarto[1]; Elena Monti[1]; Luana Toniolo[1]; Carlo Reggiani[1,2]; Emiliana Giacomello[4]; Sandra Zampieri[1,3,5]; Lisa M. Hartnell[6]; Giuseppe De Vito[1,3]; Marco Sandri[1,3]; Luigi Ferrucci[6] & Marco V. Narici[1,2,3,*]

1. Department of Biomedical Sciences, University of Padova, Padova, 35131, Italy
2. Science and Research Center Koper, Institute for Kinesiology Research, Koper, 6000, Slovenia
3. CIR-MYO Myology Center, University of Padua, 35131, Italy
4. Department of Medicine, Surgery and Health Sciences, University of Trieste, Trieste, 34149, Italy
5. Department of Surgery, Oncology and Gastroenterology, University of Padova, Padova, 35124, Italy
6. Translational Gerontology Branch Longitudinal Studies Section, National Institute on Aging, Baltimore, MD 21224, USA

*Dr Giuseppe Sirago: Department of Biomedical Sciences, University of Padova, Padova, 35131, Italy **- Email:** giuseppesirago88@gmail.com - ORCID: https://orcid.org/0000-0001-7005-8708

*Prof. Marco V. Narici: Department of Biomedical Sciences, CIR-MYO Myology Centre, University of Padova, Padova, 35131, Italy **- Email:** marco.narici@unipd.it - ORCID: https://orcid.org/0000-0003-0167-1845

**Figure S1**

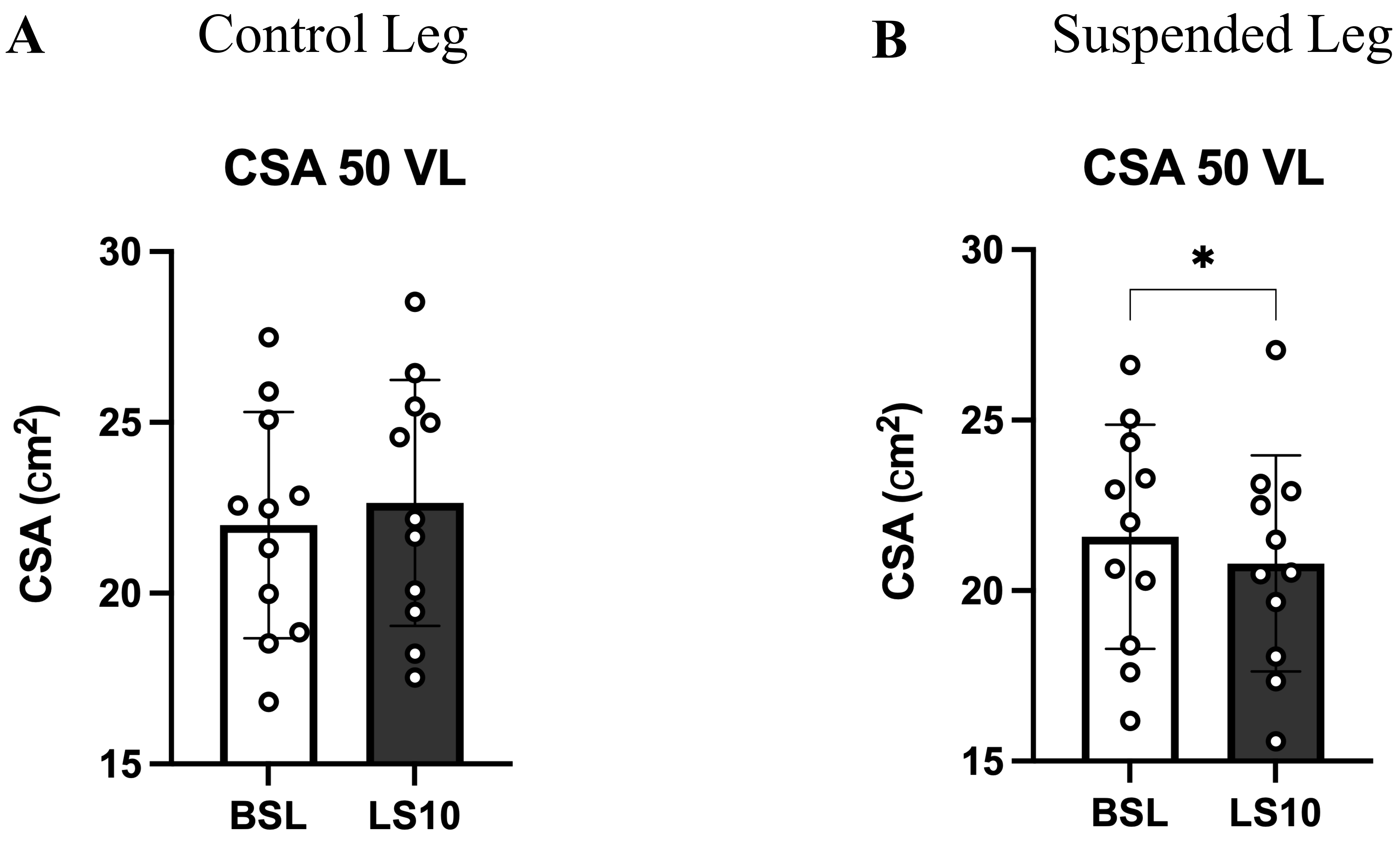

A
Control Leg
CSA 50 VL
CSA (cm²)
30
25
20
15
BSL
LS10
B
Suspended Leg
CSA 50 VL
CSA (cm²)
*
30
25
20
15
BSL
LS10

Figure S2

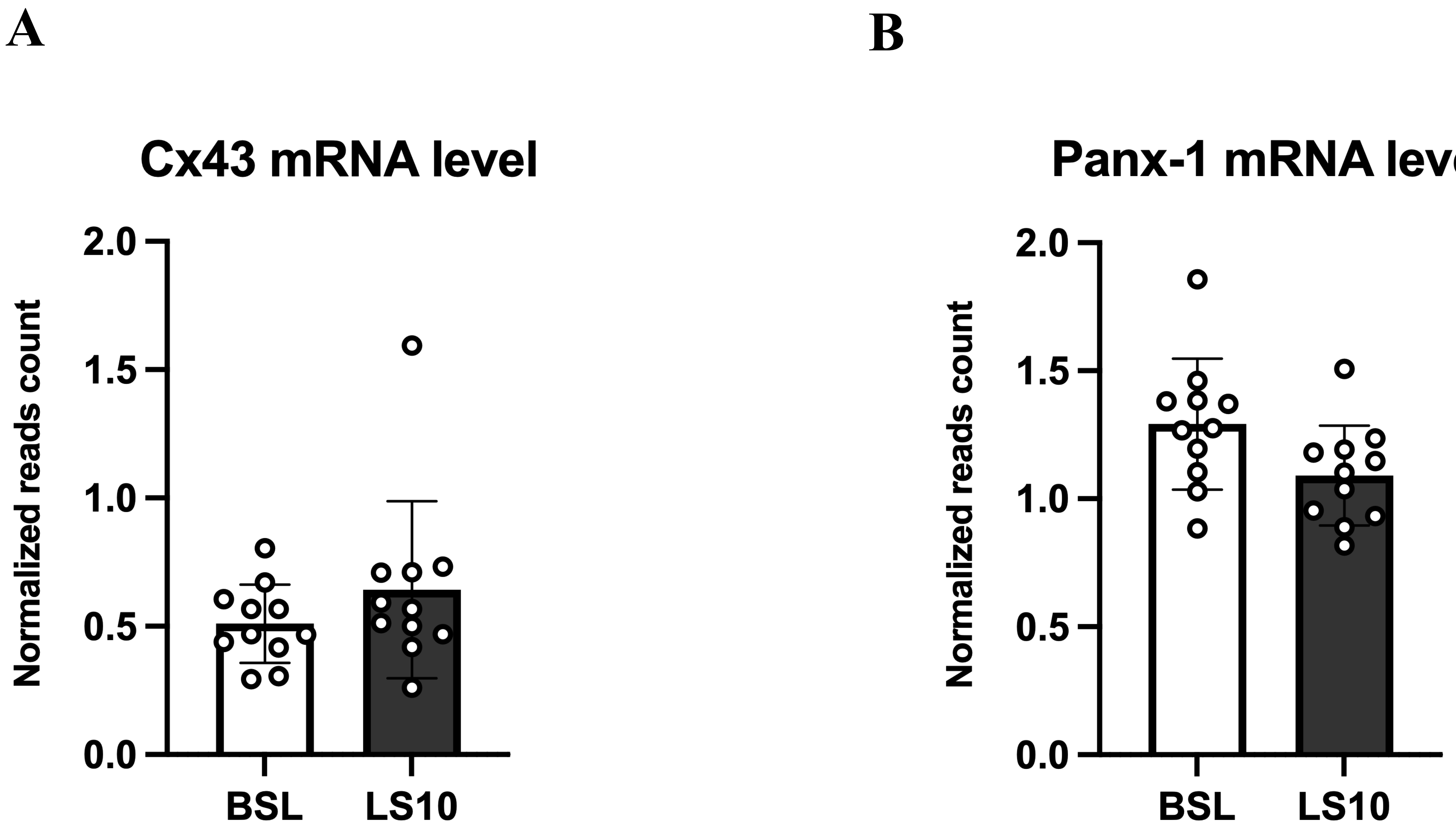
A
Cx43 mRNA level
2.0
1.5
1.0
0.5
0.0
Normalized reads count
BSL
LS10
B
Panx-1 mRNA level
2.0
1.5
1.0
0.5
0.0
Normalized reads count
BSL
LS10

**Figure S3**

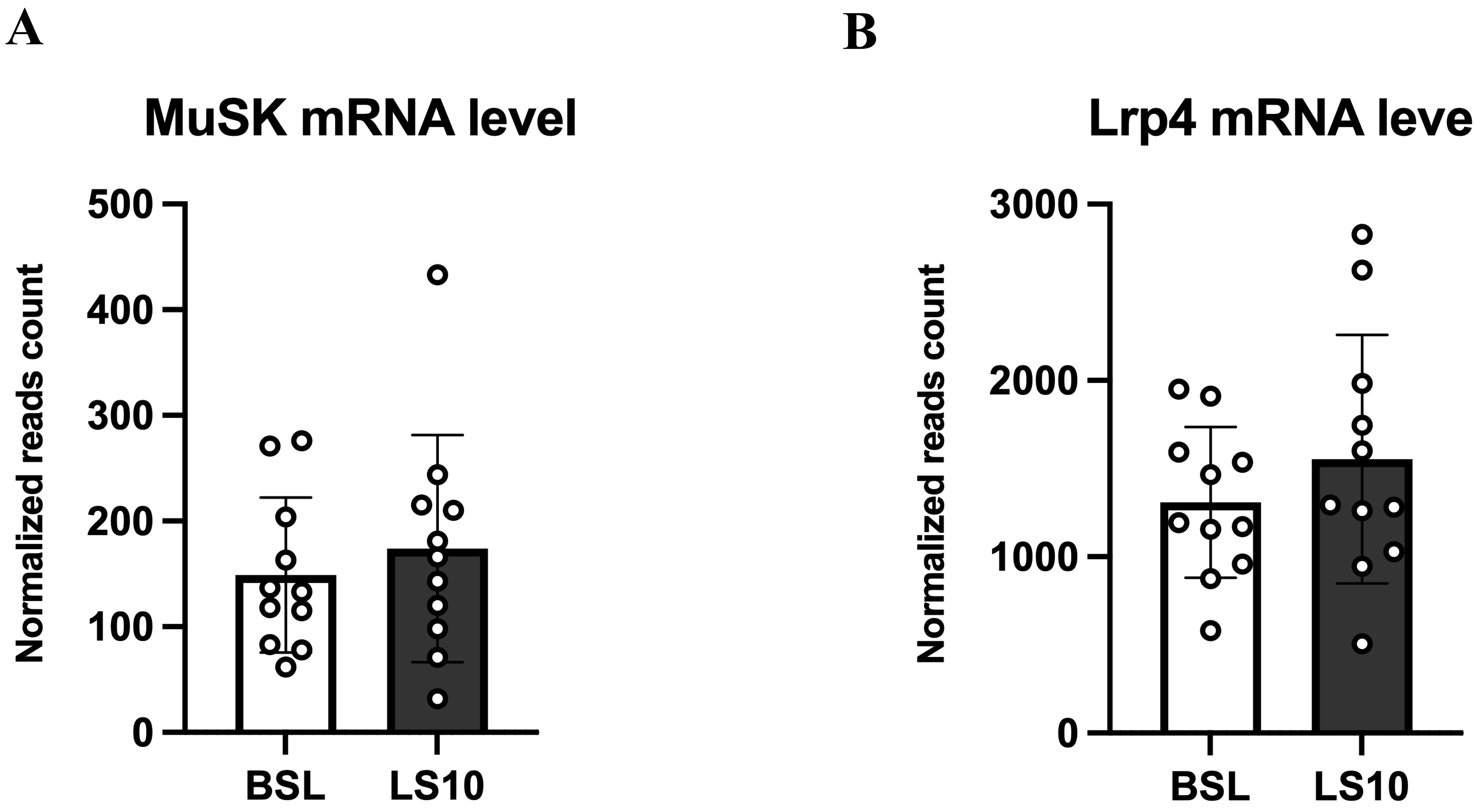
A
MuSK mRNA level
Normalized reads count
500
400
300
200
100
0
BSL
LS10
B
Lrp4 mRNA level
Normalized reads count
3000
2000
1000
0
BSL
LS10

**Figure S4**

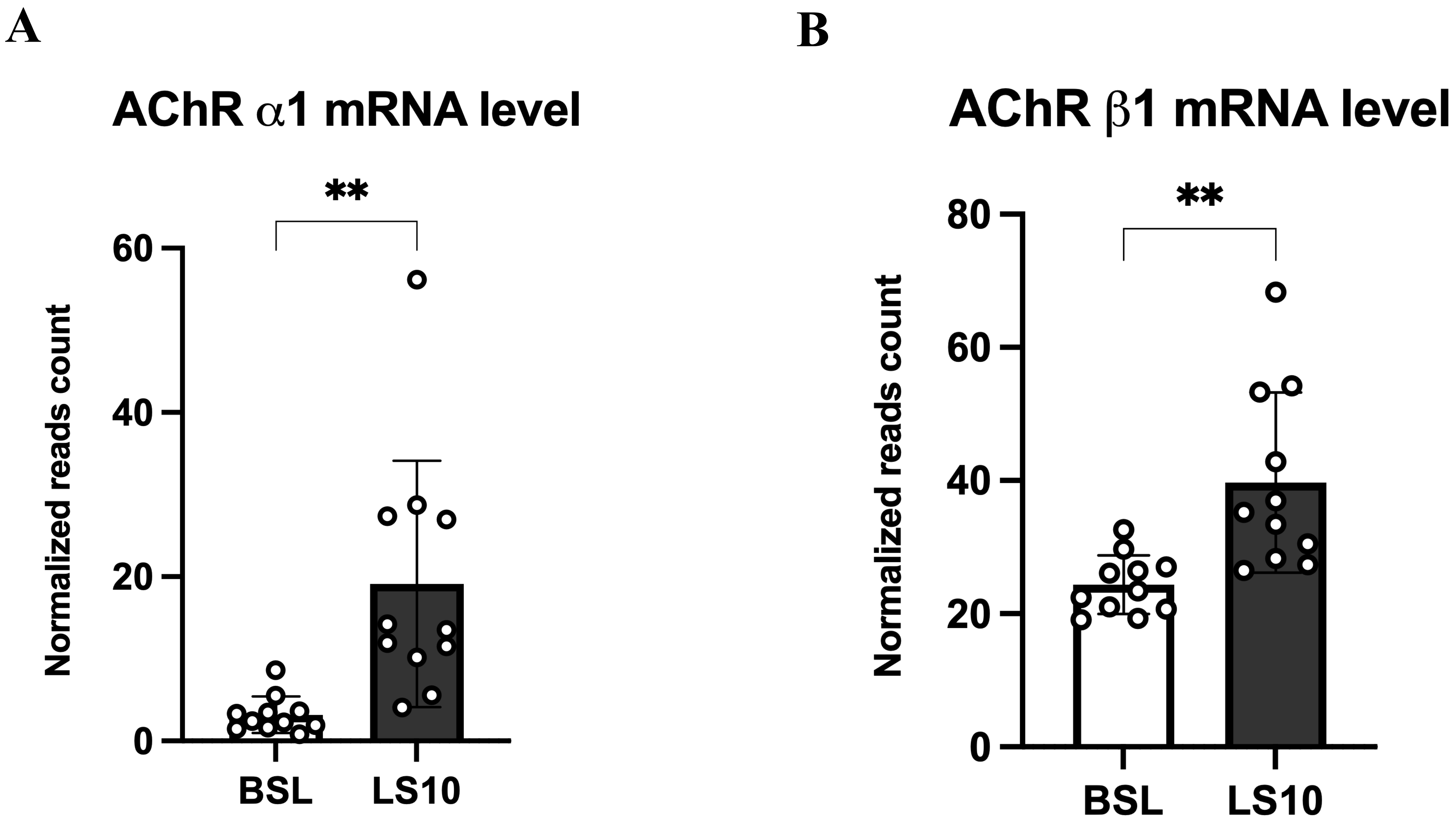
A
AChR α1 mRNA level
**
60
40
20
0
Normalized reads count
BSL
LS10
B
AChR β1 mRNA level
**
80
60
40
20
0
Normalized reads count
BSL
LS10

**Figure S5**

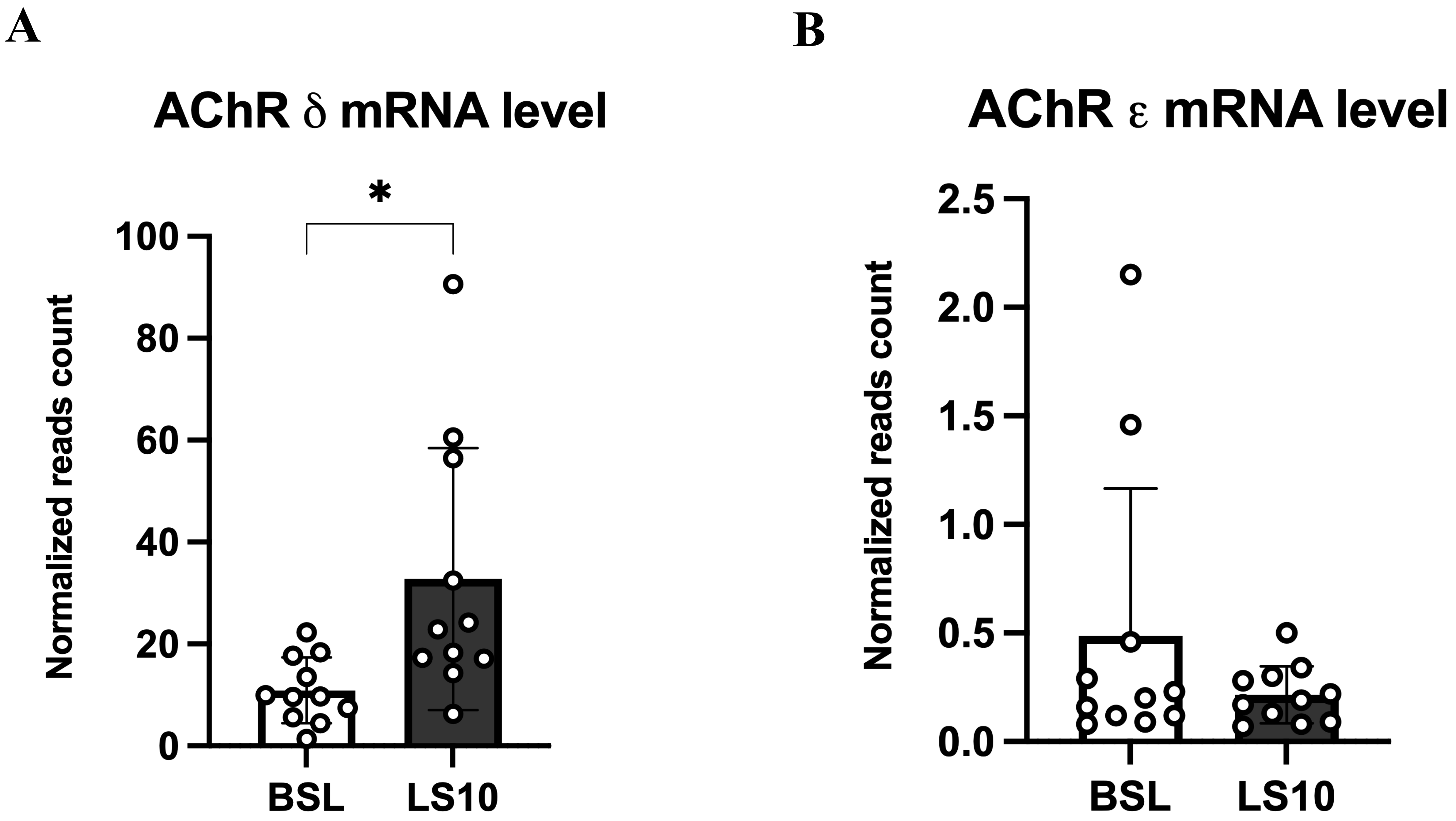
A
AChR δ mRNA level
*
100
80
60
40
20
0
Normalized reads count
BSL
LS10
B
AChR ε mRNA level
2.5
2.0
1.5
1.0
0.5
0.0
Normalized reads count
BSL
LS10

**Figure S6**

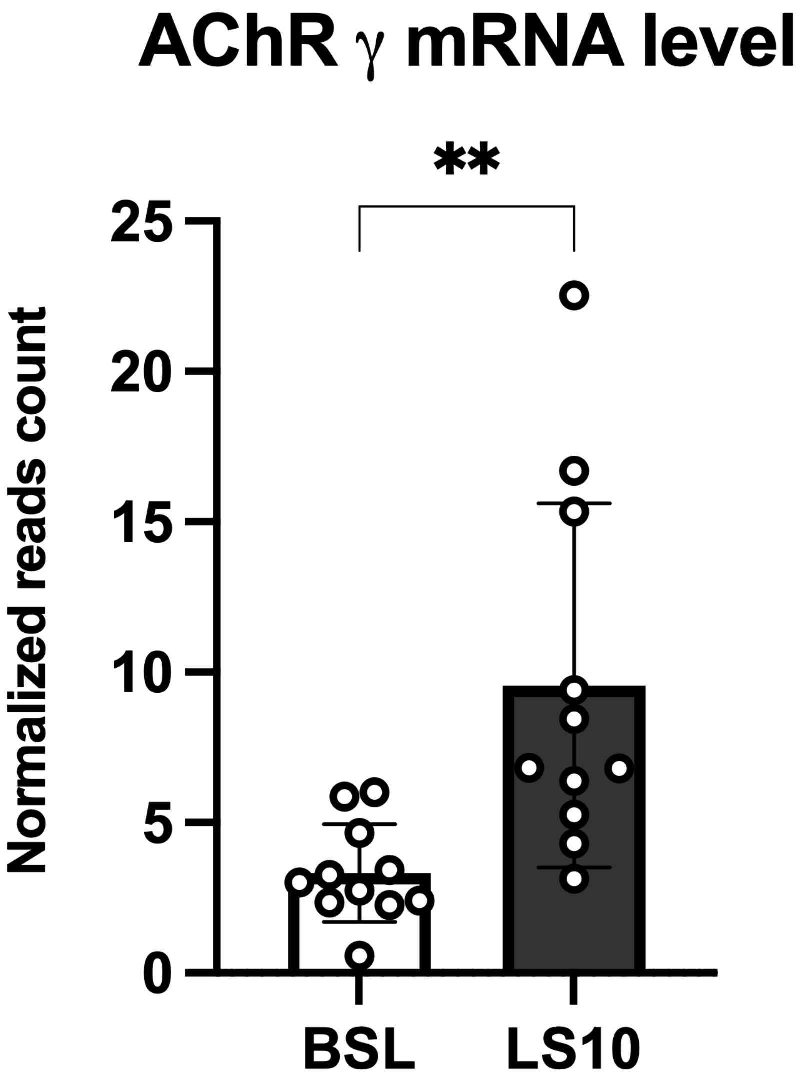

AChR γ mRNA level
**
Normalized reads count
25
20
15
10
5
0
BSL
LS10

**Figure S7**

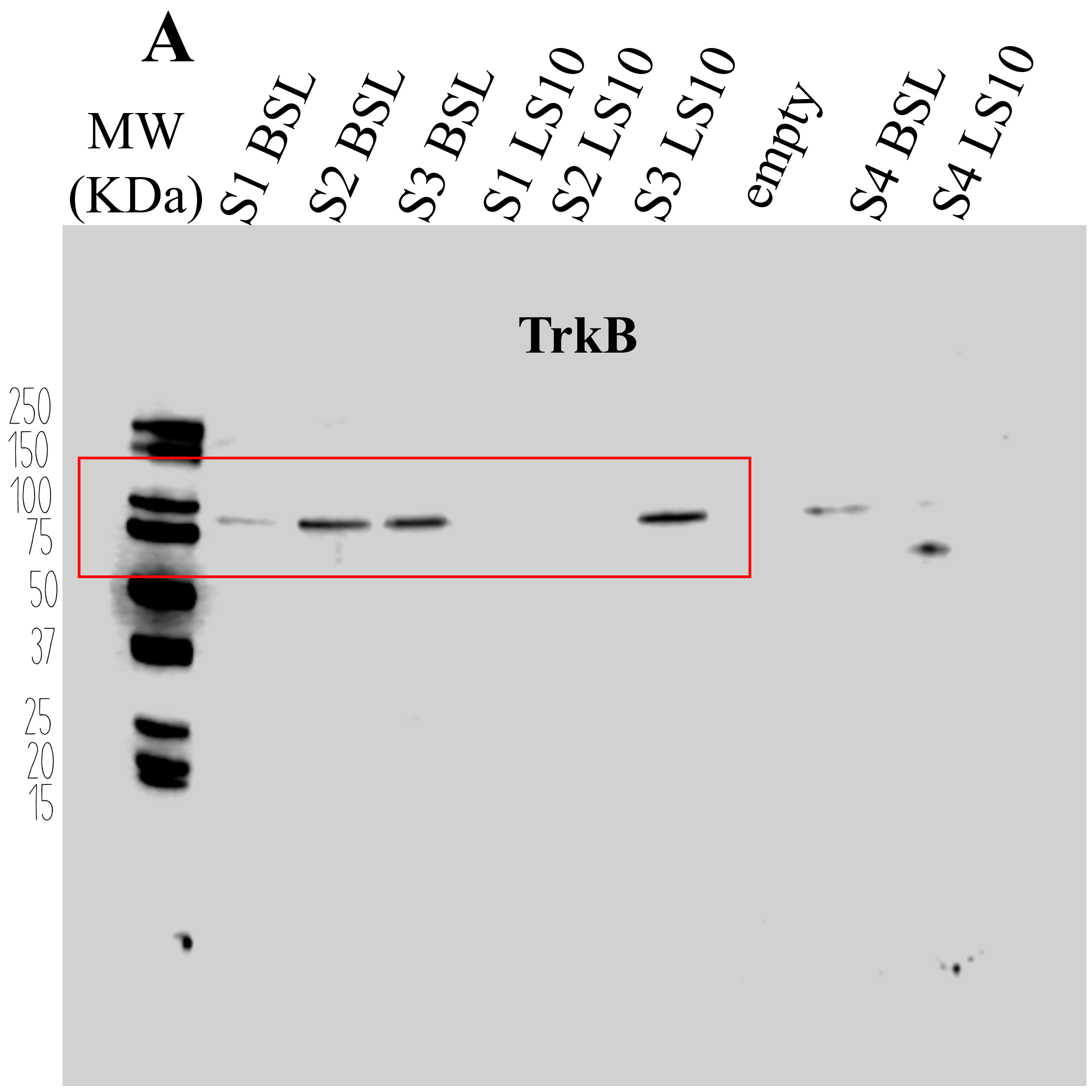
A
MW
(KDa)
S1 BSL
S2 BSL
S3 BSL
S1 LS10
S2 LS10
S3 LS10
empty
S4 BSL
S4 LS10
TrkB
250
150
100
75
50
37
25
20
15

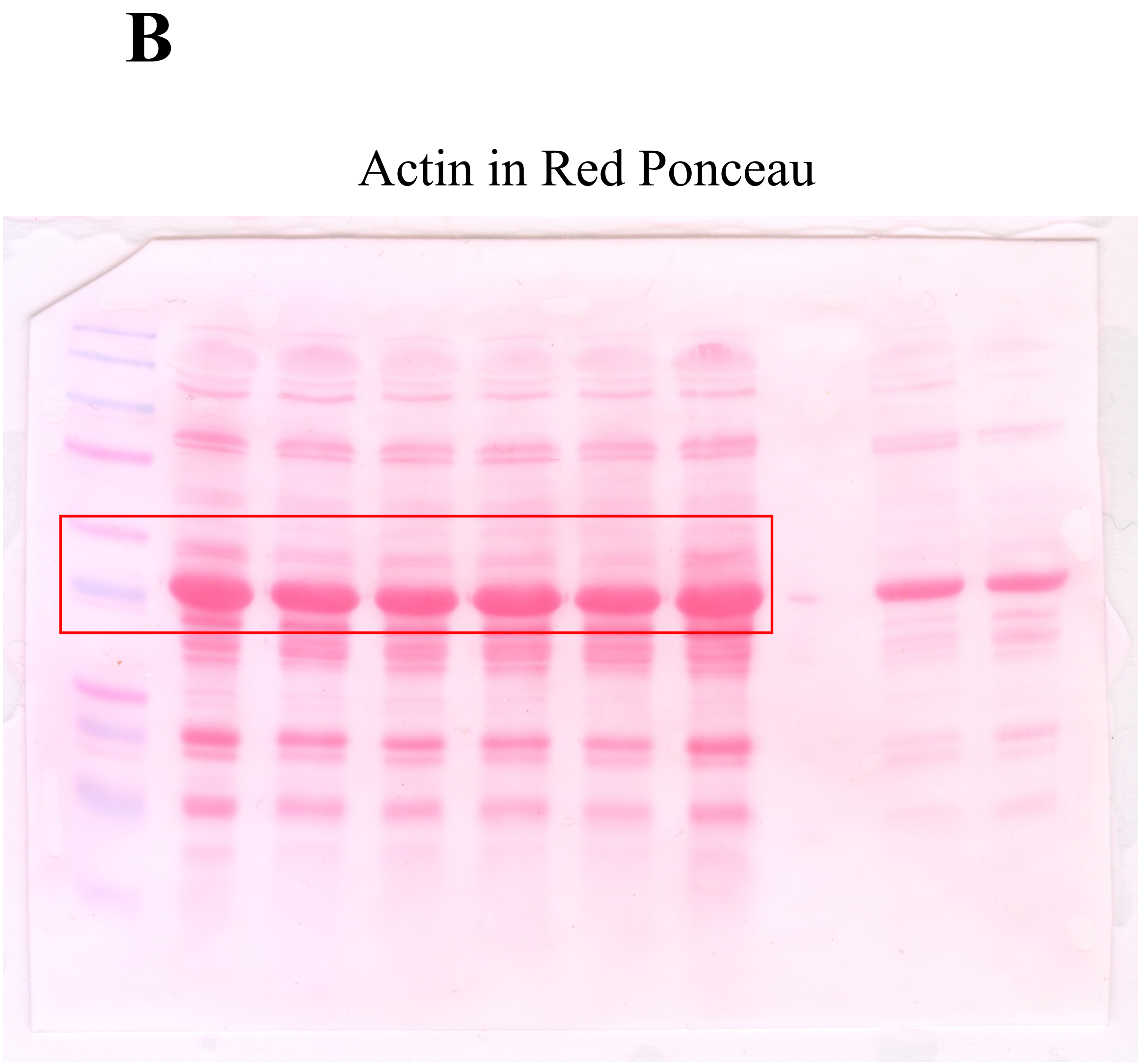
B
Actin in Red Ponceau

**Figure S8**

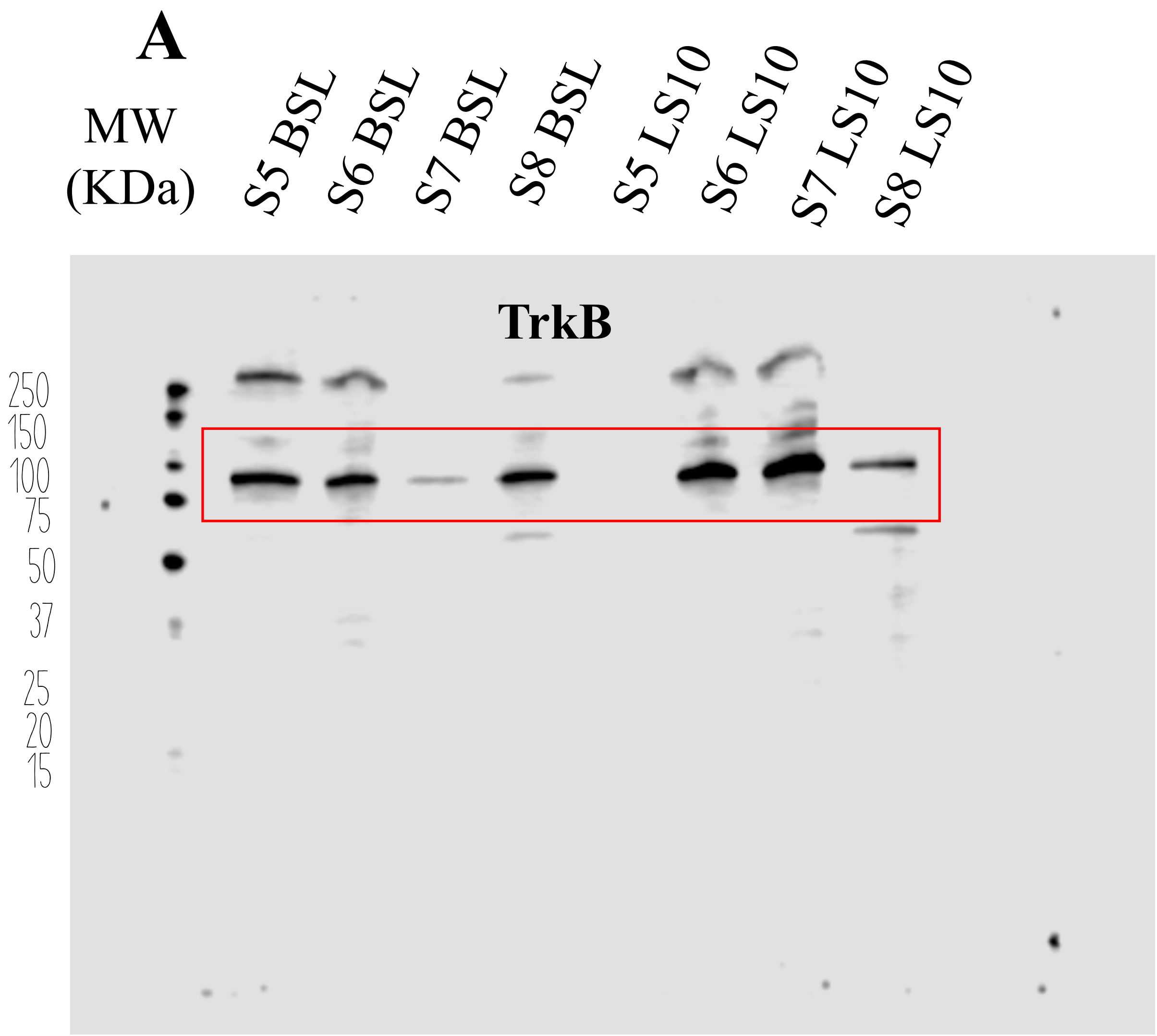
A
MW
(KDa)
S5 BSL
S6 BSL
S7 BSL
S8 BSL
S5 LS10
S6 LS10
S7 LS10
S8 LS10
TrkB
250
150
100
75
50
37
25
20
15

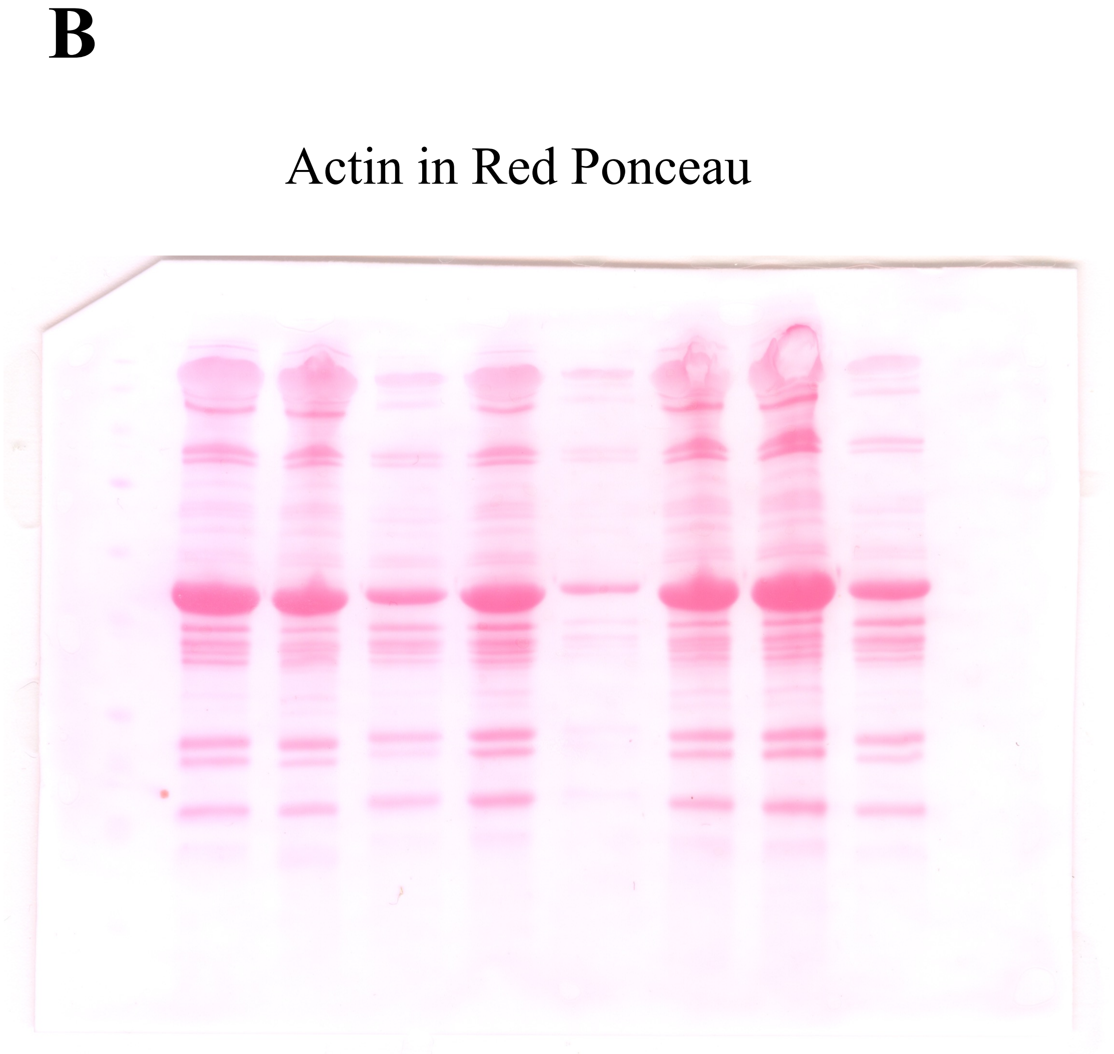
B
Actin in Red Ponceau

**Figure S9**

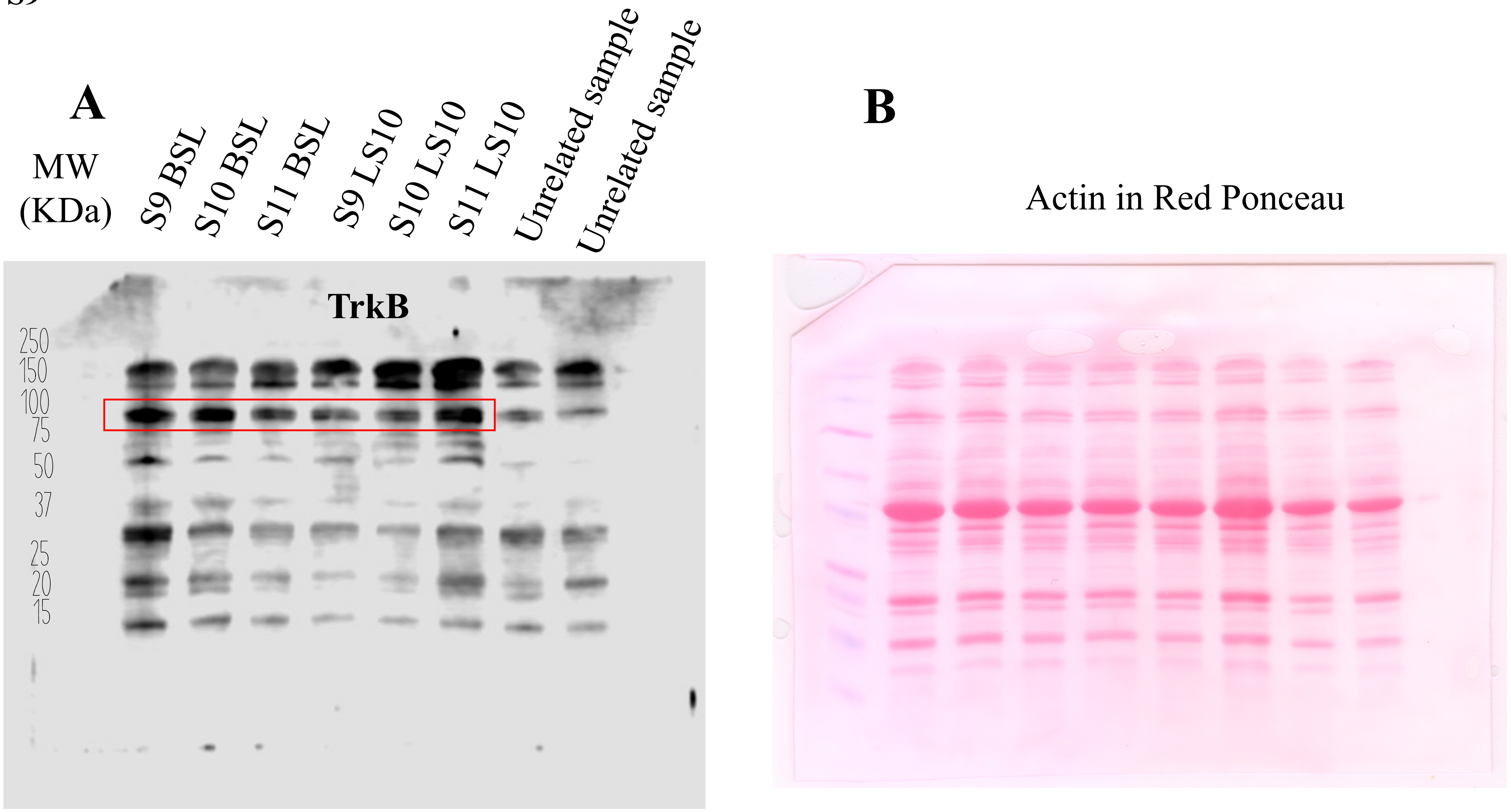

A
MW (KDa)
S9 BSL
S10 BSL
S11 BSL
S9 LS10
S10 LS10
S11 LS10
Unrelated sample
Unrelated sample
TrkB
250
150
100
75
50
37
25
20
15
B
Actin in Red Ponceau
